# Identification, Trend Analysis and Influencing Factors of Mental Health Status of the Chinese Older Adults

**DOI:** 10.3390/ijerph17218251

**Published:** 2020-11-08

**Authors:** Hongyan Yang, Jun Ma, Hongwei Hu, Fanjie Li

**Affiliations:** 1 Center for Social Security Studies, Wuhan University, Wuhan 430072, China; yhyhyang@whu.edu.cn (H.Y.); xtmajun@126.com (J.M.); 2 School of Public Administration and Policy, Renmin University of China, Haidian District, Beijing 100872, China; 3 International Education School of Zhongnan University of Economics and Law, Wuhan 430073, China; lifanjie@zuel.edu.cn

**Keywords:** mental health state, latent categorial analysis, trend, influencing factors

## Abstract

This study aimed to analyse the classification, development trends and the influencing factors of the Chinese older adults’ mental health state. Based on longitudinal data of Chinese older adults from 2005 to 2014, 2077 older adults aged 64 to 105 were included and the Latent Class Model, Latent Growth Mixture Model and Multinomial Logit models were employed in this study. We find that there are three types of mental health state of the Chinese older adults: negative, positive and contradictory; and the contradictory type could easily turn into negative or positive mental health state. There are four types of dynamic trends of mental health state: persistently negative, persistently positive, pro-negative, and pro-positive. About 40% of the older adults could maintain positive mental health state, and the pro-negative accounts for larger proportion than the pro-positive. Better economic status, good living habits, cohabitation with family members and pension coverage are beneficial for positive mental health state of the Chinese older adults. There is significant heterogeneity in the state as well as development trends of mental health of the older adults. The older adults with contradictory and negative types of mental health state should get timely psychological help to avoid turning into negative state. A series of polices are needed to promote mental health for the older adults in China.

## 1. Introduction

Health is a state of complete physical, mental and social well-being and not merely the absence of disease or infirmity [1]. The older adults is at high risk of a variety of mental health problems. According to the European MentDis_ICF65+ study, 1/2 older adults aged 65–84 had experienced mental disorder in their lifetime, 1/3 had experienced past year, and about 1/4 had a mental disorder currently [2]. In a meta-analysis, 16.5% of older adults in Western countries suffered from lifetime major depression [3]. China has the largest population of older adults in the world, and mental health of the older adults brings forward huge challenges to China. According to the Fourth Survey for Living Conditions of Urban and Rural Elderly in China, in 2015, about 36.6% of the older adults often or sometimes feel lonely [4]. One survey showed that the prevalence of depressive symptom among older adults in China was 39.96% [5], and the prevalence was 30.0% in rural China [6]. Chinese older adults are facing huge challenges in mental health, and the classification, development trends and influencing factors become increasingly urgent in academic research and policy-making in China.

A range of studies investigated the development trends of health among older adults in China [7]. The elderly Chinese’s mental health are divided into several types including positive, negative and other types [8,9]. One survey showed that 4.1% and 14.4% of the older adults in China were classified into severe type and mild type, respectively [10].

Mental health was usually measured with standardized scales and described with sum scores [11,12]. Higher score usually indicates better mental health or worse health [13]. The investigation and assessment, as well as the associated factors, are the research focus in the previous literatures. A series of studies showed that a wide variety of factors including gender, age, marital status, residence and socioeconomic status are associated with mental health state and its corresponding development trends [14,15,16]. Education and environment are regarded as significant influencing factors of mental health and its development trends [17]. Policy support and other kinds of social support are investigated to have positive impacts on mental health of older adults [18,19].

Although the previous studies have greatly advanced the understanding of basic status and influencing factors of mental health among the Chinese older adults, there were also shortcomings in previous studies. First, most of the related previous studies are based on cross-sectional data rather than balanced panel data and focus on the current state rather than transition or trajectory of older adults’ mental health. Second, previous studies mostly measured mental health state by summing the total score of each item, and the method failed to fully consider the differences between the various items and the heterogeneity of the mental health among elderly people. Therefore, studies on classification and heterogeneity of mental health trends of the Chinese older adults are insufficient and needed to be further strengthened. Third, studies on the influencing factors of mental health trends of the Chinese older adults need more attention, and the comprehensive classification analysis of mental health and its development trends as well as the influencing factors are still lacking and need to be strengthened.

Based on the above background, this present study focuses on the following topics: (1) the mental health state and its corresponding classification of the Chinese older adults; (2) the development trend analysis of mental health of the older adults as they are getting older; (3) the influencing factors of different mental health trend types of the older adults. This study aims to contribute the previous literature on the heterogeneity of current state as well as development of the Chinese older adults’ mental health state, especially on classification, development trends and the influencing factors of the older adults’ mental health state.

## 2. Materials and Methods

### 2.1. Data

Data used in this study were derived from the Chinese Longitudinal Healthy Longevity Survey (CLHLS), which was conducted jointly by Peking University and Duke University in accordance with the Declaration of Helsinki, and was approved by the Research Ethics Committees of Peking University (IRB00001052-13074) and Duke University (12-260E). The follow-up survey included 22 provinces across China and the survey area covered 1.1 billion people, accounting for 85% of China’s total population, which was the largest follow-up survey on health of the older adults in the world. The CLHLS survey randomly selected half of the counties and cities in each province and interviewed the older adults aged 65 and above who voluntarily signed written informed consent. Socio-demographic features, socioeconomic background, health status, and other information were collected in the interview (More detailed information about CLHLS can be found at: https://sites.duke.edu/centerforaging/programs/chinese-longitudinal-healthy-longevity-survey-clhls/). This study used three waves of follow-up survey data in CLHLS (2005, 2008, and 2014). The only matching condition for the merging of the three waves of data is ID (identity code), and finally the data of 2077 older adults who survived for 10 years and had complete variable information at three time points were retained. Then, on this basis, a balanced panel data is constructed. The average age of the interviewed older adults of the analysis sample was 74 years old in 2005, and males accounts for 48.9%. More comprehensive and detailed sample information will be presented in the variable description section bellow.

### 2.2. Measurement

#### 2.2.1. Dependent Variable

Traditional mental health models mainly use psychopathology (PTH) indicators, such as depression and anxiety, and the goal is to reduce or eliminate mental illness. Traditional models rely too much on negative diagnostic indicators of psychopathology, leading to the study of mental health being limited to the perspective of psychopathology, ignoring the individual’s self-recovery and self-renewal capabilities [20]. The Dual-Factor Model of Mental Health (DFM) points out that mental health is a complete state. This model breaks through the previous use of psychopathological indicators as the only measurement of mental health by incorporating the measurement indicator of subjective well-being [21]. Therefore, we define mental health as a combination status of no psychopathological symptoms and no subjective unhappiness.

Based on the definition of mental health and the accessibility of variable information in the survey data, this present study selected four items from the survey questionnaire to measure mental health of the Chinese older adults. These four items include “Do you often feel fearful or anxious”, “Do you often feel lonely and isolated”, “Do you feel the older you get, the more useless you are”, and “Are you as happy as when you were younger”. The four indicators reflect the mental health of the older adults from different perspectives: fear/anxiety and loneliness are both mental health symptoms of the older adults. The former reflects the mental pressure and anxiety, and the latter is the gap in interaction with others and the bad mood that arises when social needs are not met. The useless and unhappy emotions compared with the younger age measure the subjective happiness of the older adults. The former reflects the older adults’ judgment of self-worth and ability, and the latter reflects the mental state of the older adults. The four variables used in this study are also totally consistent with the four dimensions of Memorial University of Newfoundland Scale of Happiness (MUNSH), which was widely used to measure happiness and mental health in the world [22].

#### 2.2.2. Independent Variable

Independent variables were selected from survey data in line with previous studies which focused on health trajectory and influencing factors of the older adults [23], and these factors include individual characteristics (Gender, Age, Years of education, Residence, Self-reported Health, Activity of daily living, social participation, Appetite, Sleep quality), family characteristics (Economic status, Co-residence, Marital status, Number of children alive) and social support (Pension, Medical insurance, Accessibility to health service). These independent variables are frequently used in the previous literatures on the risk factors of mental health for the older adults [24,25].

### 2.3. Analysis Strategy

A series of analysis methods were employed in this study, and these following comprehensive analysis methods could accomplish the mission combined with latent analysis, development analysis and influencing factors analysis. The latent analysis takes the measurement errors into account and contributes to improve the measurement of dependent variables.

Latent Class Model (LCM) was used to identify different mental state categories of the Chinese older adults. The Latent Growth Mixture Model (LGMM) was used to explore the categories of mental health development trends of the Chinese older adults. Finally, Multinomial Logistic Regression model was conducted to investigate the influencing factors of the mental health trends of the older adults.

Akaike Information Criterion (AIC), Bayesian Information Criterion (BIC), Sample Size-adjusted BIC (aBIC), Entropy, LMR (Lo-Mendell-Rubin), and Bootstrap Likelihood Ratio Test (BLRT) were used to evaluate the quality of latent class models and latent growth mixture models. The smaller AIC, BIC and aBIC represents a better model fitness, and the Entropy closer to 1 means more accurate classification. Both LMR and BLRT are used to compare the fitness difference between k-1-classes and k-classes models. Significant *p*-values of LMR and BLRT show that the k-classes model is better than the k-1-classes model [26]. Mplus 7.4 software was used in this study.

## 3. Results

### 3.1. Descriptive Analysis Results

Table 1 presents the results of the description of mental health in this sample. Participants were more likely to feel that they were valueless (>0.53) and unhappy (>0.48), and less fearful/anxious (<0.26) or loneliness (<0.28).

Table 2 shows a descriptive statistic for the independent variables based on the cross-sectional data of 2005. The average age of the older adults in the sample is about 73, with 48.9% are males, and the average years of education is close to 3. Over 60% of the older adults live in rural area, and about 11.3% of the older adults are in bad self-reported health. About 2.7% of the older adults are totally functional limited in activities of daily living, and over 20% of the older adults participate social activities (social participation) in daily life. About 75% have good appetite, and about 66% have good sleep quality. About 16.6% have good economic status, and about 87.7% live with others (co-residence with others). About 62% have a spouse, and the average number of children alive is 3.96. The coverage ratios of pension and medical insurance are 24.8% and 28.4, respectively, and over 90% of the older adults could access health services in time.

### 3.2. Latent Class Analysis of Mental Health

Table 3 presents the goodness-of-fit indicators for the 1–5 classes of LCM models in different years. In general, the AIC, BIC, and aBIC values of three-classes model were the smallest (except BIC and aBIC in 2008) while the *p* values of LMR and BLRT were both significant. Thus, the three-classes model were optimally fitted based on the results.

**Figure 1:**
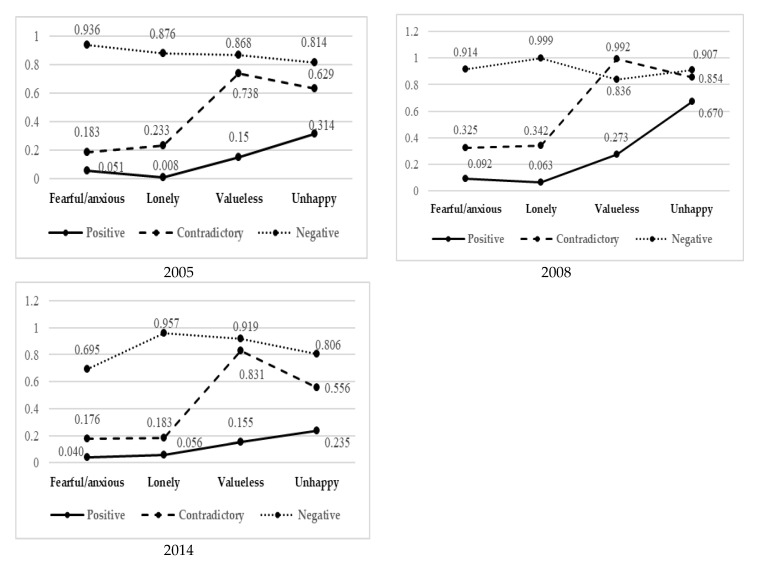
Conditional probability line chart of LCM in 2005, 2008 and 2014.

Table 4 shows the latent class probability coefficients for the three classes of mental health state of the Chinese older adults. Figure 1 showed the conditional probability line of mental health of the older adults in three years. For type 1, the older adults had weak psychological symptoms and no subjective unhappiness, so we named type 1 “positive.” For type 2, the older adults had weak psychological symptoms (felt less nervous and lonely) but had subjective unhappiness (felt more valueless and unhappy), so we named it “contradictory”. For type 3, the older adults had severe psychological symptoms and subjective unhappiness, and type 3 could be named as “negative”.

In terms of comprehensive comparison of the trends of the three types over three years, the positive and contradictory types account for higher proportion, while the negative type accounts for relatively smaller proportion.

### 3.3. The Development Trend Analysis

In order to further investigate the specific types based on mental health trends of the older adults, this study conducted a series of models with LGMM method. Positive, contradictory and negative types were coded as 1, 2 and 3, respectively. As shown in Table 5, the AIC, BIC, and aBIC values of Model 4 were the smallest while the *p* values of LMR and BLRT were both significant. Thus, four latent classes of mental health trends of the older adults are supported by the analysis results shown in Table 5.

**Figure 2:**
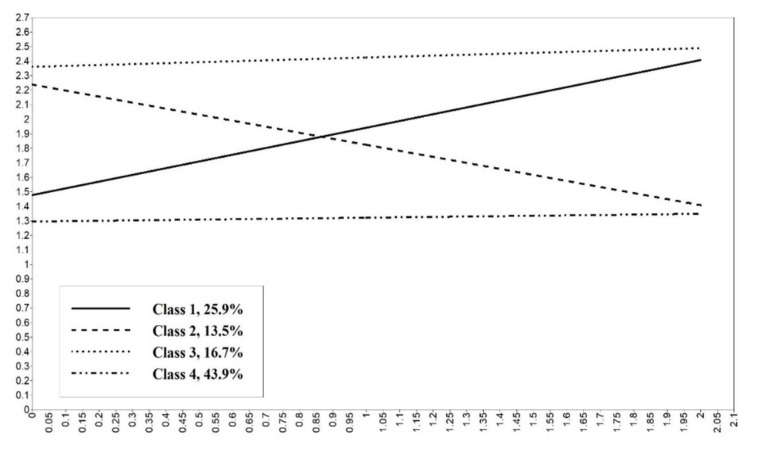
Growth trend of LGMM.

Table 6 presents the estimated results of four latent classes, while Figure 2 shows the corresponding trends of the four latent classes in one graph. Based on the comprehensive analysis of the above results, the four latent classes could be named as pro-negative (accounting for 25.9%), pro-positive (accounting for 13.5%), persistently negative (accounting for 16.7%), and persistently positive (accounting for 43.9%), respectively.

According to the analysis results, we had several further findings: (1) Persistently positive type had the highest proportion, accounting for over 40%; (2) With the increase of age, the number of pro-negative type tended to count for higher proportion than pro-positive type, and this finding further supports the above findings in the LCM model analysis that the proportion of negative type older adults increase with age; (3) the older adults with high or low levels of mental health are more likely to maintain stable mental health state, while those with middle level of mental health state are more likely to shift to positive or negative mental health state.

### 3.4. The Influence Factors of Mental Health Trend

Multinomial Logit Regression Model was employed to investigate the influencing factors of the mental health trend of the older adults, and the estimated 4 classes of mental health development trend based on LGMM analysis above were used as the dependent variable. Table 7 shows the results of Multinomial Logit Regression with the persistently negative type as the reference group in the analyses.

In terms of individual characteristics, compared with female older adults, the male older adults are more likely to have positive mental health state. The older adults with better self-reported health were more likely to maintain a positive mental health state. In addition, having good living habits, such as participation in social activities (social participation), good sleep quality and appetite, were also beneficial to maintain mental health state or shift to positive mental health state.

In terms of family characteristics, the older adults with better economic status, living with others (co-residence), having a spouse, are more likely to maintain persistently positive mental state. Compared with the poor, the older adults with middle and rich economic status are more likely to be persistently positive rather than to be the persistently negative. Compared with the older adults living alone, those living with others have higher probability of being persistently positive than the reference group (the persistently negative type).

For social support, compared with the older adults without pension, the older adults covered with pension are more likely to maintain persistently positive mental health state than the persistently negative type of older adults. The regression results showed that medical insurance and accessibility to health service have no significant impact on mental health development trends of the older adults.

## 4. Discussion

This study was aimed to investigate the classification and the development trends of mental health of the older adults, as well as the influencing factors of the development trends. This study was based on a large survey data from Chinese older adults and a series of analysis methods including LCM, LGMM and Multinomial Logit regression models. The classification and heterogeneity of mental health state of the older adults as well as the dynamic trends are the focuses in this study.

The results showed that mental health state of the older adults could be classified into three types including negative type, contradictory type and positive type. Positive and contradictory types account for much higher proportion than the negative type, while the proportion of the negative type of older adults increase fast. The above findings, especially the finding about contradictory mental health type, has taken a step forward than the previous studies which generally divided the mental health of the older adults into positive and negative types [27,28]. Those who are not detected on screening measures of psychopathology, but report diminished subjective happiness or who are “pure languishing” in life [29] need to be noticed. Meanwhile, mental health state of the contradictory type older adults was easy to change into a very negative or positive state, which further highlights the instability of contradictory type. This may be closely related to the rapid change and decline in socio-economic status after retirement which was called as “retirement syndrome” [30]. The high proportion of contradictory type accounts for emphasizes that mental health of the older adults is unstable and changeable at this stage in life course [31].

Based on the development trend analysis results, mental health trends of the Chinese older adults could be classified into four types: persistently positive, pro-positive, persistently negative and pro-negative. Compared with the previous studies which have divided physical health trends into three types (maintained function, progressive disability, and consistent disability), the findings in this present study classifies mental health development trends of the older adults into four types has deepened the understanding [32]. From the structure of four types of mental health trends, about 40% of the older adults could maintain persistently positive mental health state, and they have good self-control and life management ability, this finding is also in line with previous studies [33]. With the increase of age, the number of the older adults tending to be negative (about 25%) was larger than that tending to be positive, and this phenomenon is very noteworthy. There were several possible reasons leading to this non-negligible phenomenon in current China. First, the continuous decline in the interaction with families and society, especially the continuously weakening of primary-group contacts with family, friends and other primary groups would lead to a strong loneliness experience [34]. Due to the large-scale population migration from rural to urban areas, and the growing miniaturization of family size, as well as the declining filial piety in China, the attention and support received by the older adults are decreasing. Second, the continuous decline of physical health would further accelerate the decline of mental health status. Third, the incomplete social welfare system (especially in terms of the supply of spiritual comfort services), coupled with the diminishing of economic resources, may also cause the mental health of the older adults to become pro-negative and continue to decline [35].

Multinomial regression results show that several factors belonging to the three dimensions (individual, family and social support) exert significant impacts on the mental health trends of the Chinese older adults. In terms of personal characteristics, the male older adults is more likely to be persistently positive or pro-positive, which may be attributable to women’s lower economic status and higher social pressure as well as the relatively fragile characteristics in the traditional Confucian cultural society [36]. This study found that taking part in social activities, having good sleep and appetite was conducive to maintain positive mental health, which was in line with the activity theory emphasizing the positive effects of social activities and good living habits on mental health of the older adults through better social adaption and establishment of positive self-image [37]. In terms of family characteristics, this study found that better economic status, living with others as well as the having a spouse exert huge impacts on mental health development of the older adults, and these findings underscore the impacts of family economic status and family support on mental health of the older adults which were explained in the previous studies [38]. Better economic status and support from family (especially from the spouse) are the key supportive factors for the older adults to relieve life pressure and metal disorders, as well as to promote the positive trends of the development of mental health [39]. The older adults covered by pension have significantly higher possibility to have positive mental health development trend than those without pension system. On the one hand, this finding further supports that stable and long-term income security is beneficial for the older adults to keep positive mental health, which was in consistent with the previous literatures [40]. On the other hand, pension system in China, especially in the past two decades, has developed very rapidly, and its coverage and benefit level have been greatly improved. It has become the main economic security scheme for the older adults after retirement. Therefore, it has played an important and positive role in improving the mental health state of the older adults by reducing the incidence of poverty and enhancing their sense of economic security.

There are also limitations in this study. First, there may also be measurement biases in self-reported mental health indicators, to some extent, used in this study due to the limitation of the survey questionnaire design. Second, although the four items from CLHLS questionnaire were widely employed in the previous studies [7], a certain degree of incompleteness and ambiguity still exists within this set of measurement indicators. How to construct a comprehensive and clear mental health index of the measurement of mental health of the Chinese older adults is a crucial development direction in future research.

## 5. Conclusions

The main research conclusions were summarized as follows: firstly, based on static analysis, the mental health state of the Chinese older adults could be classified into three types (negative, contradictory and positive), and the contradictory type was easily change into negative or positive state; secondly, based on dynamic analysis, mental health trends of the older adults could be categorized into four types (persistently negative, pro-negative, persistently positive and pro-positive), and persistently positive type and pro-positive type, ranking as the top two, account for about 40% and 25%, respectively; thirdly, based on the Multinomial Logit Regression results, a series of factors (gender, social participation, economic status, living arrangement, spouse status and pension coverage) exert significant impacts on development trends of mental health of the Chinese older adults.

This study provides a range of new understandings on mental health state and the corresponding development trends and contributes a lot of policy implications. Firstly, more attention should be paid to the heterogeneity of the mental health state of the older adults, especially to the older adults in contradictory and negative mental health state. They should be regarded as the key group of policy intervention and be given timely psychological counseling, which might prevent them transforming from contradictory into negative state. Secondly, the older adults belonging to negative mental health type should be targeted in public policies, and more resources and interventions should be devoted to avoid and delay the transition into negative mental health state. Thirdly, a series of policy innovation and policy implementation (including the improvement of social participation, protection of economic security, improvement of social support, establishment of age-friendly social environment) should be treated as important policy focus for Chinese government and society [37].

## Figures and Tables

**Table 1:** Descriptive statistics of mental health of the older adults.

Items	Scale	2005 (Means)	2008 (Means)	2014 (Means)
Fearful or anxious	1 = always/often/sometimes, 0 = rarely/never	0.238	0.251	0.222
Lonely and isolated	1 = always/often/sometimes, 0 = rarely/never	0.239	0.247	0.279
Feel useless when getting older	1 = always/often/sometimes, 0 = rarely/never	0.535	0.533	0.597
Feel unhappy compared to younger age	1 = always/often/sometimes, 0 = rarely/never	0.536	0.747	0.483

**Table 2:** Descriptive statistics of independent variables (2005).

Variable	Scale and Range	%(n = 2077)/Means(SD)
Individual characteristics		
Gender	Male = 1	48.92
	Female = 0	51.08
Age	64–105, continuous variables	73.69(7.27)
Years of education	0–18, continuous variables	2.97(3.85)
Residence	Rural = 1	62.69
	Urban = 0	37.31
Self-reported health	Good = 1 Middle = 2 Bad = 3	57.15
		31.63
		11.22
Activity of daily living	Limited = 1	2.74
	Normal = 0	97.26
Social participation	Yes = 1	21.44
	No = 0	78.56
Appetite	Good and above = 1	74.68
	Else = 0	25.32
Sleep quality	Good and above = 1	66.54
	Else = 0	33.46
Family characteristics		
Economic status	Rich = 1 Middle = 2 Poor = 3	16.59
		69.14
		14.27
Co-residence	Yes = 1	87.67
	No = 0	12.33
Marital status	Having spouse = 1	61.96
	No spouse = 0	38.04
Number of children alive	0–11, continuous variables	3.96(1.73)
Social support		
Pension	Yes = 1	24.81
	No = 0	75.19
Medical insurance	Yes = 1	28.55
	No = 0	71.45
Accessibility to health service	Yes = 1	91.38
	No = 0	8.62

**Table 3:** The fit index of latent class model (LCM).

Year	Class	AIC	BIC	aBIC	Entropy	LMR	BLRT
2005	1	10,307.244	10,329.798	10,317.090	-	-	-
	2	9599.070	9649.818	9621.224	0.654	0.0000	0.0000
	3	9569.261	9648.202	9603.723	0.561	0.0000	0.0000
	4	9577.905	9685.040	9624.676	0.458	0.4950	0.5000
	5	9587.905	9723.234	9646.984	0.620	0.5634	1.0000
2008	1	9890.891	9913.446	9900.737	-	-	-
	2	9228.250	9278.998	9250.404	0.661	0.0000	0.0000
	3	9228.029	9306.971	9262.491	0.671	0.0745	0.0789
	4	9237.728	9344.863	9284.498	0.608	0.7081	1.0000
	5	9247.728	9383.056	9306.806	0.667	0.1421	1.0000
2014	1	10,345.513	10,368.068	10,355.360	-	-	-
	2	9558.638	9609.386	9580.792	0.643	0.0000	0.0000
	3	9518.727	9597.668	9553.189	0.567	0.0000	0.0000
	4	9524.215	9631.350	9570.985	0.475	0.1040	0.1200
	5	9534.215	9669.543	9593.293	0.512	0.9190	1.0000

Note. AIC, Akaike Information Criterion; BIC, Bayesian Information Criterion; aBIC, Adjusted BIC; LMR, Lo-Mendell-Rubin; BLRT, Bootstrap Likelihood Ratio Test.

**Table 4:** Probability and proportion of latent class model of mental health.

Items	2005 (n = 2077)			2008 (n = 2077)			2014 (n = 2077)		
	Positive	Contradictory	Negative	Positive	Contradictory	Negative	Positive	Contradictory	Negative
Fearful/anxious	0.051	0.183	0.936	0.092	0.325	0.914	0.040	0.176	0.695
Lonely	0.008	0.233	0.876	0.063	0.342	0.999	0.056	0.183	0.957
Valueless	0.150	0.738	0.868	0.273	0.992	0.836	0.155	0.831	0.919
Unhappy	0.314	0.629	0.814	0.670	0.854	0.907	0.235	0.556	0.806
Proportion (%)	39.86	46.61	13.53	51.13	34.86	14.01	36.73	42.13	21.14

**Table 5:** The fitness indexes of LGMM.

Model	AIC	BIC	aBIC	Entropy	LMR	BLRT	Class probability (%)
Model 1	13,479.551	13,524.661	13,499.244	-	-	-	100
Model 2	13,397.146	13,459.171	13,424.223	0.524	0.0001	0.0000	38.5/61.5
Model 3	13,322.520	13,401.462	13,356.983	0.615	0.0000	0.0000	42.6/6.7/50.7
Model 4	13,248.482	13,344.339	13,290.329	0.613	0.0000	0.0000	25.9/13.5/16.7/43.9
Model 5	13,254.482	13,367.255	13,303.713	0.666	0.5000	1.0000	0.0/13.5/25.9/43.9/16.7

**Table 6:** The results of LGMM.

Classes	Means	Est.	S.E.	Est./S.E.	*p*
Pro-negative (25.9%)	intercept	1.477	0.067	22.043	0.000
	Slope	0.465	0.046	10.037	0.000
Pro-positive (13.5%)	intercept	2.238	0.072	31.149	0.000
	Slope	−0.414	0.056	−7.399	0.000
Persistently negative (16.7%)	intercept	2.361	0.080	29.353	0.000
	Slope	0.065	0.056	1.161	0.246
Persistently positive (43.9%)	intercept	1.295	0.037	34.631	0.000
	Slope	0.027	0.026	1.040	0.298

Note. Est., Estimate; S.E., Standard Error.

**Table 7:** Multinomial Logit Regression Analysis.

Variables	Pro-Negative		Pro-Positive		Persistently Positive	
	B	Exp(B)	B	Exp(B)	B	Exp(B)
Gender	0.120	1.127	0.354 *	1.425	0.529 ***	1.697
Age	−0.002	0.998	0.023	1.023	0.008	1.008
Years of education	0.006	1.006	0.011	1.011	0.025	1.025
Residence	−0.117	0.889	−0.017	0.983	−0.262	0.769
Self-reported health						
Middle	0.060	1.061	0.079	1.083	0.325	1.384
Good	0.556 **	1.744	0.241	1.273	0.947 ***	2.579
Activity of daily living	−0.352	0.704	−0.359	0.699	−0.633	0.531
Social participation	0.206	1.228	0.402 *	1.495	0.340 *	1.405
Appetite	0.322 **	1.380	0.530 ***	1.699	0.373 **	1.452
Sleep quality	0.359 **	1.432	0.166	1.181	0.658 ***	1.931
Economic status General Rich						
	0.163	1.177	0.148	1.159	0.550 ***	1.733
	0.581 **	1.788	0.305	1.357	0.941 ***	2.564
Co-residence	0.359	1.432	0.399	1.491	0.429 **	1.536
Marital status	0.390 **	1.477	0.437 **	1.547	0.555 ***	1.743
Number of children alive	0.016	1.016	−0.028	0.972	−0.029	0.971
Pension	0.746 ***	2.109	0.431	1.539	0.796 ***	2.218
Medical insurance	0.007	1.007	−0.080	0.923	0.088	1.092
Accessibility to health service	0.207	1.230	0.053	1.055	0.334	1.396
Constants	−1.185	0.306	−3.398 ***	0.033	−2.666 ***	0.070
Log likelihood	−2460.9418					
Chi-square Statistics	330.78 ***					
Pseudo R^2^	0.0630					

Note: * *p* < 0.10, ** *p* < 0.05, *** *p* < 0.01.

## References

[1] Saracci R. (1997). The world health organisation needs to reconsider its definition of health. BMJ.

[2] Andreas S., Schulz H., Volkert J., Dehoust M., Sehner S., Suling A., Ausín B., Canuto A., Crawford M., da Ronch C. (2017). Prevalence of mental disorders in elderly people: The European MentDis_ICF65+ study. Br. J. Psychiatry.

[3] Volkert J., Schulz H., Härter M., Wlodarczyk O., Andreas S. (2013). The prevalence of mental disorders in older people in Western countries—A meta-analysis. Ageing Res. Rev..

[4] Dang J.W. (2018). Survey Report on the Living Conditions of China’s Urban and Rural Older Persons.

[5] Yu J., Li J., Cuijpers P., Wu S., Wu Z. (2012). Prevalence and correlates of depressive symptoms in Chinese older adults: A population-based study. Int. J. Geriatr. Psychiatry.

[6] Yang Z., Chen R., Hu X., Ren X.H. (2017). Factors that related to the depressive symptoms among elderly in urban and rural areas of China. Zhonghua Liu Xing Bing Xue Za Zhi = Zhonghua Liuxingbingxue Zazhi.

[7] Liang Y., Lu P. (2014). Medical insurance policy organized by Chinese government and the health inequity of the elderly: Longitudinal comparison based on effect of New Cooperative Medical Scheme on health of rural elderly in 22 provinces and cities. Int. J. Equity Health.

[8] Sun X., Lucas H., Meng Q., Zhang Y. (2010). Associations between living arrangements and health-related quality of life of urban elderly people: A study from China. Qual. Life Res..

[9] Yin K.L., He J.M., Fu Y.F. (2013). Mental well-being (111–132). Positive Mental Health: Measurement, Prevalence, and Correlates in a Chinese Cultural Context.

[10] Yu E.S.H., Liu W.T., Levy P., Zhang M.-Y., Katzman R., Lung C.-T., Wong S.-C., Wang Z.-Y., Qu G.-Y. (1989). Cognitive Impairment Among Elderly Adults in Shanghai, China. J. Gerontol..

[11] Guo Y.-Q., Zhang C.-C., Huang H., Zheng X., Pan X.-J., Zheng J.-Z. (2016). Mental health and related influencing factors among the empty-nest elderly and the non-empty-nest elderly in Taiyuan, China: A cross-sectional study. Public Health.

[12] Kruk K.E., Reinhold S. (2014). The effect of children on depression in old age. Soc. Sci. Med..

[13] Drydakis N. (2015). The effect of unemployment on self-reported health and mental health in Greece from 2008 to 2013: A longitudinal study before and during the financial crisis. Soc. Sci. Med..

[14] Butterworth P., Rodgers B., Windsor T. (2009). Financial hardship, socio-economic position and depression: Results from the PATH Through Life Survey. Soc. Sci. Med..

[15] Luppa M., Sikorski C., Luck T., Ehreke L., Konnopka A., Wiese B., Weyerer S., König H.-H., Riedel-Heller S. (2012). Age- and gender-specific prevalence of depression in latest-life—Systematic review and meta-analysis. J. Affect. Disord..

[16] Joutsenniemi K. (2006). Living arrangements and mental health in Finland. J. Epidemiol. Community Heal..

[17] Martin L.G., Zimmer Z., Hurng B.-S. (2011). Trends in late-life disability in Taiwan, 1989–2007: The roles of education, environment, and technology. Popul. Stud..

[18] Bozo Ö., Toksabay N.E., Kürüm O. (2009). Activities of Daily Living, Depression, and Social Support Among Elderly Turkish People. J. Psychol..

[19] Koizumi Y., Awata S., Kuriyama S., Ohmori K., Hozawa A., Seki T., Matsuoka H., Tsuji I. (2005). Association between social support and depression status in the elderly: Results of a 1-year community-based prospective cohort study in Japan. Psychiatry Clin. Neurosci..

[20] Alan C. (2004). Positive Psychology: The Science of Happiness and Human Strengths.

[21] Suldo S.M., Shaffer E.J. (2008). Looking Beyond Psychopathology: The Dual-Factor Model of Mental Health in Youth. Sch. Psychol. Rev..

[22] Han W.-J., Shibusawa T. (2015). Trajectory of physical health, cognitive status, and psychological well-being among Chinese elderly. Arch. Gerontol. Geriatr..

[23] Lee H.-L., Huang H.-C., Lee M.-D., Chen J.H., Lin K.-C. (2012). Factors affecting trajectory patterns of self-rated health (SRH) in an older population—A community-based longitudinal study. Arch. Gerontol. Geriatr..

[24] Haug M., Belgrave L.L., Gratton B. (1984). Mental Health and the Elderly: Factors in Stability and Change Over Time. J. Heal. Soc. Behav..

[25] Cullati S., Rousseaux E., Gabadinho A., Courvoisier D.S., Burton-Jeangros C. (2014). Factors of change and cumulative factors in self-rated health trajectories: A systematic review. Adv. Life Course Res..

[26] Nylund K.L., Asparouhov T., Muthén B.O. (2007). Deciding on the Number of Classes in Latent Class Analysis and Growth Mixture Modeling: A Monte Carlo Simulation Study. Struct. Equ. Model. A Multidiscip. J..

[27] Cutrona C., Russell D., Rose J. (1986). Social support and adaptation to stress by the elderly. Psychol. Aging.

[28] Heidrich S.M., Ryff C.D. (1993). The Role of Social Comparisons Processes in the Psychological Adaptation of Elderly Adults. J. Gerontol..

[29] Keyes C.L.M. (2002). The Mental Health Continuum: From Languishing to Flourishing in Life. J. Heal. Soc. Behav..

[30] de Vries M.K. (2003). The Retirement Syndrome. Eur. Manag. J..

[31] Hsu H.-C. (2010). Trajectory of Life Satisfaction and its Relationship with Subjective Economic Status and Successful Aging. Soc. Indic. Res..

[32] Yu H.-W., Chen D.-R., Chiang T.-L., Tu Y.-K., Chen Y.-M. (2015). Disability trajectories and associated disablement process factors among older adults in Taiwan. Arch. Gerontol. Geriatr..

[33] Lightsey O.R., Maxwell D.A., Nash T.M., Rarey E.B., McKinney V.A., Lightsey J.O.R. (2011). Self-Control and Self-Efficacy for Affect Regulation as Moderators of the Negative Affect–Life Satisfaction Relationship. J. Cogn. Psychother..

[34] Peters G.R., Hoyt D.R., Babchuk N., Kaiser M., Iijima Y. (1987). Primary-Group Support Systems of the Aged. Res. Aging.

[35] Melchiorre M.G., Chiatti C., Lamura G., Torres-Gonzales F., Stankunas M., Lindert J., Ioannidi-Kapolou E., Barros H., Macassa G., Soares J.F.J. (2013). Social Support, Socio-Economic Status, Health and Abuse among Older People in Seven European Countries. PLoS ONE.

[36] Shi M.Q., Zhou X.Z. (2013). Research on mental health and the demand of spiritual care service for urban elderly—A case of Shanghai. World Survey Res..

[37] Albrecht R. (1953). Relationships of Older People with Their Own Parents. Marriage Fam. Living.

[38] Frey B.S., Stutzer A. (2002). What can economists learn from happiness research?. J. Econ. Lit..

[39] Blanchflower D.G., Oswald A.J. (2004). Well-being over time in Britain and the USA. J. Public Econ..

[40] Zhang W., Liu G. (2007). Childlessness, Psychological Well-being, and Life Satisfaction Among the Elderly in China. J. Cross-Cultural Gerontol..

